# Combining Robot-Assisted Gait Training and Non-Invasive Brain Stimulation in Chronic Stroke Patients: A Systematic Review

**DOI:** 10.3389/fneur.2022.795788

**Published:** 2022-05-02

**Authors:** Federica Bressi, Alex Martino Cinnera, Giovanni Morone, Benedetta Campagnola, Laura Cricenti, Fabio Santacaterina, Sandra Miccinilli, Loredana Zollo, Stefano Paolucci, Vincenzo Di Lazzaro, Silvia Sterzi, Marco Bravi

**Affiliations:** ^1^Physical Medicine and Rehabilitation Unit, Campus Bio-Medico University of Rome, Rome, Italy; ^2^Istituto di Ricovero e Cura a Carattere Scientifico (IRCCS) Santa Lucia Foundation, Rome, Italy; ^3^Unit of Advanced Robotics and Human-Centred Technologies, Campus Bio-Medico University of Rome, Rome, Italy; ^4^Unity of Neurology, Neurophysiology, Neurobiology, Department of Medicine, Campus Bio-Medico University of Rome, Rome, Italy

**Keywords:** robotics, transcranial direct current stimulation, chronic stroke, robot-assisted, exoskeleton, transcranial magnetic stimulation, TMS, NIBS

## Abstract

Gait impairment is one of the most common disorders of patients with chronic stroke, which hugely affects the ability to carry out the activities of daily living and the quality of life. Recently, traditional rehabilitation techniques have been associated with non-invasive brain stimulation (NIBS) techniques, which enhance brain plasticity, with the aim of promoting recovery in patients with chronic stroke. NIBS effectiveness in improving gait parameters in patients with chronic stroke has been in several studies evaluated. Robotic devices are emerging as promising tools for the treatment of stroke-related disabilities by performing repetitive, intensive, and task-specific treatments and have been proved to be effective for the enhancement of motor recovery in patients with chronic stroke. To date, several studies have examined the combination of NIBS with robotic-assisted gait training, but the effectiveness of this approach is not yet well established. The main purpose of this systematic review is to clarify whether the combination of NIBS and robot-assisted gait training may improve walking function in patients with chronic stroke. Our systematic review was conducted according to the preferred reporting items for systematic reviews and meta-analyses (PRISMA) guidelines. Studies eligible for review were identified through PubMed/MEDLINE, Embase, Scopus, and PEDro from inception to March 15, 2021, and the outcomes considered were gait assessments. Seven studies were included in the qualitative analysis of this systematic review, with a total population of 186 patients with chronic stroke. All studies specified technical characteristics of robotic devices and NIBS used, with high heterogeneity of protocols. Methodological studies have shown a significantly greater improvement in walking capacity recorded with 6MWT. Finally, research studies have highlighted a positive effect on walking recovery by combination of robot-assisted gait training with non-invasive brain stimulation. Furthermore, future studies should identify the best characteristics of the combined therapeutic protocols.

**Systematic Review Registration:**

CRD42021244869.

## Introduction

Stroke is the leading cause of disability worldwide. In Europe, in 2017, there were 1.12 million strokes (1). In the United States, more than 795,000 have a stroke each year, and about 610,000 of these are first strokes (2). Motor impairment is the most common consequence of stroke, which can be regarded as loss or limitation of function in muscle control or movement in an arm and a leg on one side of the body (3). Motor impairment of the lower limb, frequently present among patients with stroke, often results in gait disorders, hugely impacting the ability to carry out the activities of daily living and the quality of life (4, 5). Despite the efficacy of a large variety of physiotherapy interventions in improving functional outcomes in all post-stroke phases (6, 7), 6 months after stroke, more than 30% of survivors cannot walk independently (3). For this reason, it is necessary to develop novel neurorehabilitation treatments to minimize long-term disability (8).

Based on this, in recent decades, new technologies have been introduced and coupled with physical therapy with the aim of enhancing motor recovery of the lower limbs and walking ability. Among these, the use of robotic devices is emerging as promising tools for the treatment of stroke-related disabilities; robotic devices allow repetitive, intensive, and task-specific treatments that have been proved to be effective for promoting motor recovery in patients with chronic stroke (9). Robotics devices for walking rehabilitation can be classified according to the way they assist a patient's lower limbs. Morone et al. (10) distinguished two groups of these devices: exoskeletons that move the hip, knee, and ankle joints during the gait phases, and end-effector robots that move only the feet, often positioned on a support that imposes a specific trajectory, simulating the stance and swing phases during gait training.

A recent meta-analysis has shown that people who receive electromechanical-assisted gait training in combination with physiotherapy after stroke are more likely to achieve independent walking than people who receive gait training without these devices (11). Moreover, several studies have shown that robotic-assisted gait training (RAGT) led to functional improvement even in the chronic phase of stroke (12, 13). Parallelly, the potential of rehabilitation techniques has been enhanced by the use of non-invasive brain stimulation (NIBS), which facilitates neuroplasticity. Transcranial magnetic stimulation (TMS) and transcranial direct current stimulation (tDCS) are the two most common types of NIBS, which, by modulating cortical excitability, may induce plastic changes in the brain (14). tDCS and TMS techniques seem to be effective in enhancing motor performance in patients with stroke (15–17). NIBS effectiveness in improving gait parameters has been proved by several randomized controlled trials on patients with chronic stroke (18, 19). Although tDCS in association with neurorobotics was suggested as feasible, the efficacy is currently under debate (20).

In this regard, NIBS and neurorobotics training or functional task training (21) have been combined with the aim of maximizing the enhancement of cortical plasticity. Therefore, RAGT will help improve the walking ability of patients with chronic stroke. Optimization of training protocol, promoting active participation of patients, and the use of add-on techniques, such as tDCS (22), may be considered to enhance the effects of RAGT in patients with chronic stroke. However, to date, the efficacy of NIBS, combined with robotic-assisted gait training, has not been well established. Therefore, the main purpose of this systematic review is to clarify whether the combination of NIBS and robot-assisted gait training may improve walking function in patients with chronic stroke.

## Materials and Methods

The systematic review was conducted in three steps in accordance with the preferred reporting items for systematic reviews and meta-analyses (PRISMA) guidelines (23) 1. literature search; 2. data extraction, and 3. critical appraisal. The review protocol has been registered on PROSPERO (registration ID: CRD42021244869) (International Prospective Register of Systematic Reviews).

### Literature Search

An online systematic search was performed using the most popular electronic databases: PubMed/MEDLINE, Embase, Scopus, Web of Science (WOS), Ebsco, and PEDro from inception to March 15, 2021. We used the combination of medical subject heading (MeSH) terms and free-text terms and were adjusted according to specification of each database. The search strategy is shown in Appendix 1 in Supplementary Material. The language of publication was limited to English. We selected all design studies that use NIBS coupled with RAGT. Three reviewers (B.C., L.C., and M.B.) independently and synchronously screened the titles and abstracts to identify potentially eligible articles. In case an article was only selected by one reviewer, the three reviewers discussed whether to include a study in the full-text analysis. A fourth reviewer (A.M.C.) was consulted in case a consensus between the first three reviewers was not reached. Subsequently, all the reviewers independently assessed the full text of the selected articles. After the selection of eligible studies, data were extracted, included the first author's full-name, year of publication, type of study, number of intervention and the control group, characteristics of population (e.g., mean age, prevalence of male), characteristics of stroke, type of exoskeleton and NIBS used, duration and follow-up, and outcomes used.

### Data Extraction

In agreement with the PRISMA guidelines (23), we reported the results using the PICOST-DS tool, focusing on the participant, intervention, comparator, outcomes, time, setting, study design (24). The PICOST-DS model was adopted to conduct an evidence-based practice literature search and, consequently, to enhance the quality of health education interventions and programs (25) (Table 1).

**Table 1:** The PICOTS-SD model.

**P-Participants**	Adult (> 18 years) Affected with chronic (> 6 months) stroke
**I-Intervention**	EXOSKELETON associated with Non Invasive Brain Stimulation (NIBS)
**C-Comparator**	Presence of a control group with characteristics comparable to the experimental group
**O-Outcomes**	Focus on mobility index
**T-Time**	No limits of time were imposed
**S-Setting**	Rehabilitation both inpatients and outpatients
**SD-Study design**	All design studies

*PICOTS-SD = participant, intervention, comparator, outcomes, time, setting, study design*.

### Critical Appraisal

The methodological quality was assessed using the version two of the Cochrane risk-of-bias tool for randomized trials (RoB 2) (26) to evaluate the quality of randomized controlled trials (RCTs). Instead, we used methodological index for non-randomized studies (MINORS) (27) to examine non-RCTs studies. The RoB 2 is structured into a fixed set of domains of bias, focusing on different aspects of trial design, conduct, and reporting. Within each domain, a series of questions (“signaling questions”) aim to elicit information about features of the trial that is relevant to risk of bias. A proposed judgment about the risk of bias arising from each domain is generated by an algorithm based on answers to the signaling questions. Judgment can be “low” or “high” risk of bias, or can express “ome concerns” (26). The MINORS index includes 12 items that are scored 0 (not reported), 1 (reported but inadequate) or 2 (reported and adequate), the global ideal score being 16 for non-comparative studies and 24 for comparative studies.

## Results

### Data Synthesis

**Figure 1:**
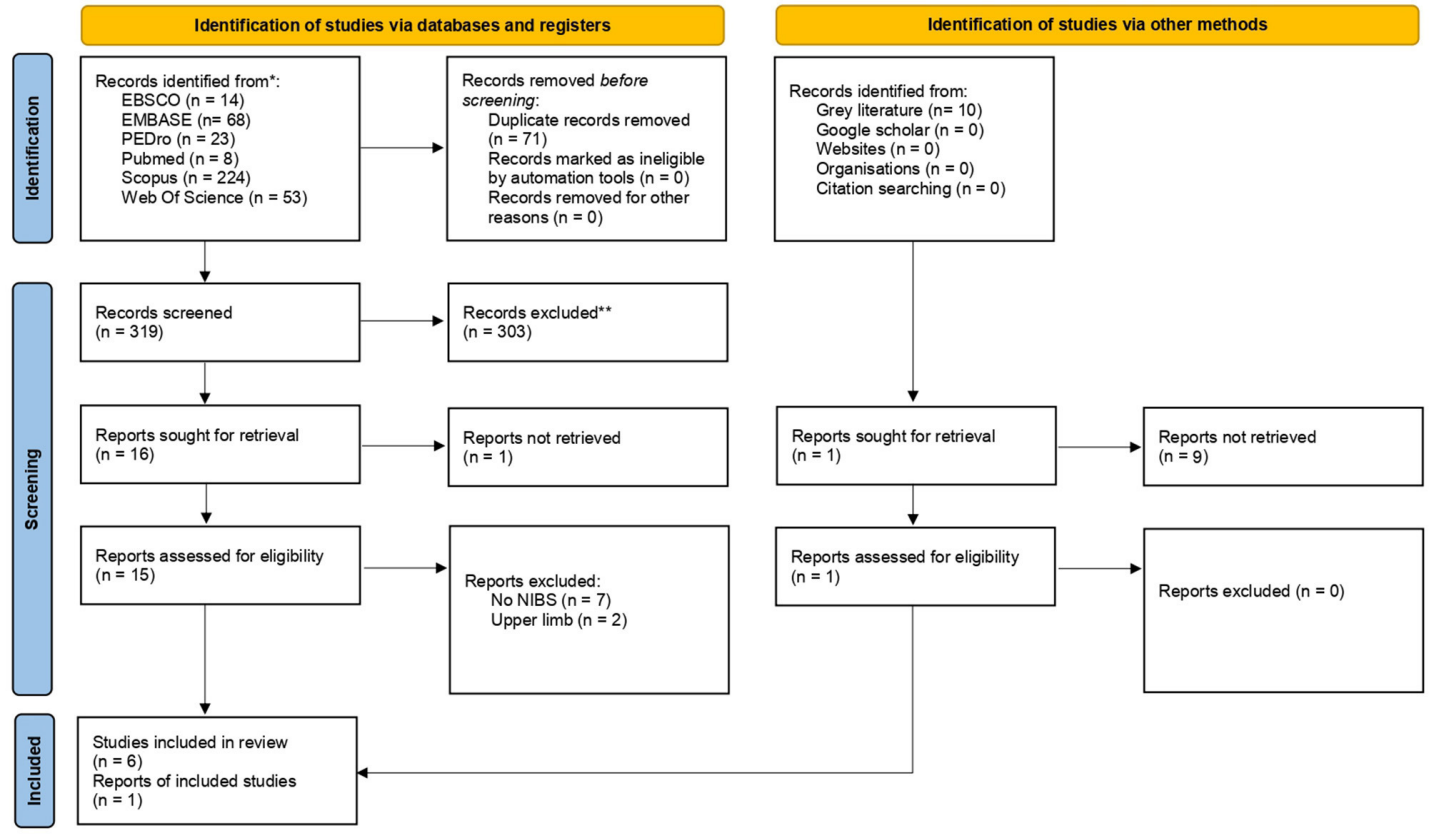
PRISMA 2020 flow diagram for new systematic reviews which included searches of databases, registers and other sources. From Page et al. (23).

A flow diagram of the research is reported in Figure 1. We found 319 records through the research method. After screening of the title and abstract, 303 articles were excluded because they did not meet our inclusion criteria (Table 1). Therefore, 17 articles were assessed for eligibility. After full-text reading, 7 studies were included in the qualitative analysis of this systematic review (20, 22, 28–32). The characteristics of the included studies are summarized in Table 2. It was revealed that studies were published between 2011 (22) and 2020 (28). Except for one retrospective clinical study (28), all the included studies were RCTs, and four of these studies were designed as pilot RCT (22, 29, 30, 32) and one as feasibility RCT (20).

**Table 2:** Characteristics of included studies.

**Author**	**Study design**	**Group 1**			**Group 2**			**Group 3**			**Additional Therapy**	**Outcome**	**Follow-up**	**Drop out**
		**Robotic device**	**Type of NIBS and additional stimulation**	**Intervention frequency**	**Robotic device**	**Type of NIBS and additional stimulation**	**Intervention frequency**	**Robotic device**	**Type of NIBS and additional stimulation**	**Intervention frequency**				
Danzl et al. (20)	Feasibility of an RCT study	Lokomat (Hocoma Inc, Zurich, Switzerland)	tDCS	3 days/week, for 4 weeks (20–40 mins)	Lokomat (Hocoma Inc, Zurich, Switzerland)	Sham tDCS	3 days/week, for 4 weeks (20–40 mins)	–	–	–	–	10MWT; BBS, FAC, SIS-16, TUG, qualitative data	After treatment, 1 month after treatment	2 during follow-up evaluation
Geroin et al. (22)	Pilot RCT	Gait Trainer GT1 (Reha-Stim, Berlin, Germany)	tDCS	5 days/week for 2 weeks (50-mins)	Gait Trainer GT1 (Reha-Stim, Berlin, Germany)	Sham tDCS	5 days/week for 2 weeks (50-mins)	–	–	5 days/week for 2 weeks (50-mins)	30 mins of LL muscle strengthening and JM	6MWT, 10MWT, FAC, MAS, MI leg subscore, R-MI, ST gait parameters	After treatment, 1 month after treatment	–
Naro et al. (28)	Retrospective clinical study	Lokomat®Pro (Hocoma Inc, Zurich, Switzerland)	bi-hemispheric dstDCS on-RAGT	6 days/week, for 8 weeks (60-mins)	Lokomat®Pro (Hocoma Inc, Zurich, Switzerland)	bi-hemispheric dstDCS post-RAGT	6 days/week, for 8 weeks (60-mins)	Lokomat®Pro (Hocoma Inc, Zurich, Switzerland)	bi-hemispheric dstDCS pre-RAGT	6 days/week, for 8 weeks (60-mins)	physical rehabilitation program (1 h) daily	6MWT, 10MWT, FAC, FIM, MI, MEP, Tinetti Scale	After treatment; 1 month and 3 months after treatment	2: 1 during treatment and 1 during follow-up evaluation
Picelli et al. (29)	Pilot RCT	G-EO System Evolution (Reha Technology, Olten, Switzerland)	Anodal tDCS+ Sham tsDCS	5 days/week for 2 weeks (20 mins)	G-EO System Evolution (Reha Technology, Olten, Switzerland)	Sham tDCS + cathodal tsDCS	5 days/week for 2 weeks (20 mins)	G-EO System Evolution (Reha Technology, Olten, Switzerland)	Anodal tDCS+ Cathodal tsDCS	5 days/week for 2 weeks (20 mins)	–	6MWT, FAC, MAS, MI leg subscore, R-MI, ST gait parameters	After treatment; 2 weeks and 4 weeks after treatment	–
Picelli et al. (29)	Pilot RCT	G-EO System Evolution (Reha Technology, Olten, Switzerland)	Cathodal tDCS on the CL cerebellar hemisphere + cathodal tsDCS	5 days/week for 2 weeks (20 mins)	G-EO System Evolution (Reha Technology, Olten, Switzerland)	Anodal tDCS over the IL cerebral hemisphere + cathodal tsDCS	5 days/week for 2 weeks (20 mins)	–	–	–	–	6MWT, AS, FAC, MI, ST gait parameters	After treatment; 2 weeks and 4 weeks after treatment	–
Picelli et al. (31)	RCT	G-EO System Evolution (Reha Technology, Olten, Switzerland)	Cathodal tDCS over the CL cerebellar hemisphere + cathodal tsDCS	5 days/week for 2 weeks (20 mins)	G-EO System Evolution (Reha Technology, Olten, Switzerland)	Cathodal tDCS over the IL cerebellar hemisphere + cathodal tsDCS	5 days/week for 2 weeks (20 mins)	–	–	–	–	6MWT, AS, FAC, MI STgait parameters	After treatment; 2 weeks and 4 weeks after treatment	1 during follow-up evaluation
Seo et al. (30)	Pilot RCT	Walkbot_S (Walkbot_S; P&S Mechanics, Seoul, Republic of Korea)	tDCS	5 days/week for 2 weeks (45 mins)	Walkbot_S (Walkbot_S; P&S Mechanics, Seoul, Republic of Korea)	Sham tDCS	5 days/week for 2 weeks (45 mins)	–	–	–	–	6MWT, 10MWT, BBS, FAC; FMA-LE, MRC	After treatment; 4 weeks after treatment	4 during follow-up evaluation

*6MWT, 6-min walking test; 10MWT, 10-meter walking test; AS, Ashworth scale; BBS, Berg balance scale; CL, contralesional; FAC, functional ambulatory category; FIM, functional independence measure; FMA-LE, Fugl-Meyer assessment of lower extremity; IL, ipsilesional; JM, joint mobilization; LL, lower limb; MAS, modified Ashworth scale; MEP, motor-evoked potentials; MI leg subscore, Motricity index leg subscore; MRC, medical research council scale; RCT, randomized control trial; R-MI, rivermead motricity index; SIS-16, stroke impact scale 16; ST, spatiotemporal; tDCS, transcranial direct current stimulation; tsDCS, transpinal direct current stimulation; TUG, timed up and go*.

### Population

The studies included a total population of 186 patients with chronic stroke (72 females) aged ≥ 18 years. The sample size of the studies ranged from 8 (20) to 40 (31), and mean patients' age in the studies ranged from 61 (29) to 72 (28) years. According to the inclusion criteria, time from the stroke onset is ≥ 6 months for all selected study: mean time between the stroke onset and the start of treatment ranged between 10 (28) and 152.5 months (30) (see Table 3).

**Table 3:** Characteristics of the participants.

**Study**	**Group 1**		**Group 2**		**Group 3**	
	**Characteristics of participants**	**Characteristics of stroke**	**Characteristics of participants**	**Characteristics of stroke**	**Characteristics of participants**	**Characteristics of stroke**
	N.participants (F:M) Age (mean ± SD)	Type of stroke (I:H) Months after stroke (mean ± SD) Affected hemisphere (L:R) Lesion localization (%)				
	N.participants (F:M) Age (mean ± SD)	Type of stroke (I:E) Months after stroke (mean ± SD) Affected hemisphere (L:R) Lesion localization				
	N.participants (F:M) Age (mean ± SD)	Type of stroke (I:E) Months after stroke (mean ± SD) Affected hemisphere (L:R) Lesion localization				
Danzl et al. (20)	4 (1:3) 64.75 ± 12.87	Chronic stroke (2:2) Months after stroke 57.3 ± 55.3 Affected hemisphere: (4:0) lesion localization: N/A	4 (3:1) 70.75 ± 9.65	Chronic stroke (4:0) Months after stroke: 26.7 ± 5.1 Affected hemisphere: (4:0) lesion localization: N/A	–	–
Geroin et al. (22)	10 (2:8) 63.6 ± 6.7	Chronic stroke (10:0) Months after stroke	10 (4:6) 63.3 ± 6.4	Chronic stroke (10:0) Month after stroke: 26.7 ± 5.1 Affected hemisphere: N/A Lesion localization: cortical 50%; subcortical 20%; mixed 30%	10 (1:9) 61.1 ± 6.3	Chronic stroke (10:0) Month after stroke: 26.9 ± 5.0 Affected hemisphere N/A Lesion localization: cortical 30%; subcortical 40%; mixed 30%
Naro et al. (28)	9 (5:4) 68 ± 4	Chronic stroke (9:0) Month after stroke: 10 ± 2 Affected hemisphere (11:4) Lesion localization: cortical 33%; large subcortical 11%; cortical-subcortical 44%; lacunar 11%	15 (9:6) 66 ± 5	Chronic stroke (15:0) Month after stroke: 11 ± 3 Affected hemisphere (11:4) Lesion localization: cortical 40%; large subcortical 13%; cortical-subcortical 40%; lacunar 7%	13 (8:5) 72 ± 4	Chronic stroke (13:0) Month after stroke: 8 ± 2 Affected hemisphere (10:3) Lesion localization: cortical 31%; large subcortical 15%; cortical-subcortical 38%; lacunar 15%
Picelli et al. (29)	10 (3:7) 64.8 ± 6.0	Chronic stroke (10:0) Month after stroke: 61.3 ± 29.3 Affected hemisphere: N\A Lesion localization: cortical 40%; subcortical 30%; mixed 30%	10 (2:8) 61.0 ± 7.2	Chronic stroke (10:0) Month after stroke: 54.8 ± 32.9 Affected hemisphere: N\A Lesion localization: cortical 40%; subcortical 20%; mixed 40%	10 (3:7) 62.8 ± 11.8	Chronic stroke (10:0) Month after stroke: 51.9 ± 41.1 Affected hemisphere: N\A Lesion localization: cortical 30%; subcortical 30%; mixed 40%
Picelli et al. (29)	10 (3:7) 62.6 ± 8.25	Chronic stroke (10:0) Month after stroke: 67.1 ± 46.75 Affected hemisphere: N\A Lesion localization: cortical 30%; subcortical 40%; mixed 30%	10 (4:6) 62.8 ± 11.81	Chronic stroke (10:0) Month after stroke: 51.9 ± 41.15 Affected hemisphere: N\A Lesion localization: cortical 40%; subcortical 30%; mixed 30%	–	–
Picelli et al. (31)	20 (10:10) 63.9 ± 10.6	Chronic stroke (20:0) Month after stroke: 66.4 ± 48.8 Affected hemisphere: N\A Lesion localization: cortical 30%; subcortical 40%; mixed 30%	20 (9:11) 65.6 ± 9.7	Chronic stroke (20:0) Month after stroke: 61.7 ± 40.1 Affected hemisphere: N\A Lesion localization: cortical 40%; subcortical 30%; mixed 30%	–	–
Seo et al. (30)	10 (3:7) 62.9 ± 8.9	Chronic stroke (8:2) Month after stroke: 152.5 ± 122.8 Affected hemisphere (2:8) Lesion localization: N\A	11 (2:9) 61.1 ± 8.9	Chronic stroke (5:6) Month after stroke: 75.5 ± 83.4 Affected hemisphere (5:6) Lesion localization: N\A	–	–

*F, female; H, Hemorrhagic stroke; I, ischemic stroke; L, left; M, male; R, right*.

### Intervention

The approach used in the intervention group was combined robot-assisted gait training and NIBS stimulation, with the latter performed before training (20, 28, 30), during training (22, 28, 29, 31, 32), or after training (28) (complete treatment characteristics are reported in Table 2).

### Robotic Treatment Characteristics

All studies specified technical characteristics of robot devices (i.e., the model, the manufacturing company, and the country of production): G-EO System Evolution (Reha Technology, Olten, Switzerland) was the only one used in more than one study (29, 31, 32). Other robotic devices utilized were Gait Trainer GT1 (Reha-Stim, Berlin, Germany) (22); Lokomat (Hocoma Inc., Zurich, Switzerland) (20);; Lokomat®Pro (Hocoma Inc., Zurich, Switzerland) (26), and Walkbot_S (P&S Mechanics, Seoul, Republic of Korea) (30). Intervention frequency ranged from 3 times a week (20) to 6 times a week (28), with a mean duration session of 33 min (minimum of 20 min; maximum of 60 min); however, more than half of the training programs was carried out 5 times a week for 2 weeks, and every session lasted 20 min (22, 29, 31, 32). Two studies added a traditional therapy to the robotic one; Geroin et al. (22) associated lower limbs muscle strengthening and joint mobilization exercises with exoskeleton therapy, and Naro et al. (28) added 1 h of a physical rehabilitation program. Conventional therapy and exoskeleton therapy with sham NIBS were mostly provided for the control group. One study (30) was sponsored by the manufacturer of the gait robot. For other studies, it was either explicitly declared that the work was not supported by any grant from the public or private sector or that there was nothing to disclose financially (20, 22, 28), or information funding was not available (29, 31, 32).

### NIBS Characteristics

All studies included in the systematic review used tDCS treatment, however, with high heterogeneity of protocols. All the studies set the intensity of stimulation at 2 mA with the exception of one using 1.5 mA (22). The electrode positioning area was specified for each study, following the 10–20 international EEG system (33). The cortical motor area was the most used site of stimulation, with exception of two studies (31, 32), in which the position of the electrodes varied according to the study group analyzed. Regarding electrodes, five studies (20, 22, 28–30) used a rectangular electrode, while the remaining two studies (31, 32) used circular electrodes. In addition, the Cathodal and Anodal electrodes had the same size—only Danzl et al. (20) – used an anodal electrode smaller than the cathodal one (25 vs. 35 cm^2^). The duration of stimulation ranged from 7 to 20 min, five out seven studies used 20 min of stimulation, while Geroin et al. (22) used 7 min of stimulation, and Naro et al. (28) used 10 min of stimulation. The technical data of the stimulator (i.e., name, the manufacturing company, and the country of production) were available for all the studies other than Danzl et al. (20) and Naro et al. (28) (complete NIBS characteristics are available in Table 4).

**Table 4:** Characteristics of NIBS.

**Study**	**Stimulator** **Model (industry, country of production)**	**Anodal electrode** **Position; Size (cm^**2**^)**	**Cathodal electrode** **Position; Size (cm^**2**^)**	**Intensity**	**Duration (min)**	**Intervention in groups**
Danzl et al. (20)	Not specified	CMA controlling leg; 25 cm^2^	Supraorbitally 35 cm^2^	2 mA	20 min Before training	Group 1: anodal+cathodal tDCS Group 2: sham anodal+sham cathodal tDCS
Geroin et al. (22)	Phyaction 787 (Uniphy, The Netherlands)	Affected CMA presumed controlling leg 35 cm^2^	Controlesional orbit 35 cm^2^	1.5 mA	7 min During training	Group 1: anodal+cathodal tDCS Group 2: sham anodal+sham cathodal tDCS Group 3: no NIBS
Naro et al. (28)	Not specified	Affected M1 (C3 or C4 position) 35 cm^2^	Unaffected M1 (C3 or C4 position) 35 cm^2^	2 mA	10 min Group 1: during training Group 2: after training Group 3: before training	Group 1: anodal+cathodal dstDCS Group 2: anodal+cathodal dstDCS Group 3: anodal+cathodal dstDCS
Picelli et al. (29)	Phyaction 787 (Uniphy, The Netherlands)	Affected M1 (C3 or C4 position) 35 cm^2^	Unaffected orbit 35 cm^2^	2 mA	20 min During training	Group 1: anodal tDCS+sham tsDCS Group 2: sham tDCS+cathodal tsDCS Group 3: anodal tDCS +cathodal tsDCS
Picelli et al. (29)	Starstim®, (Neuroelectrics, Spain)	tcDCS: Controlesional buccinator muscle 12,56 cm^2^ tDCS: Lesioned M1, Cz position 12,56 cm^2^ tsDCS:shoulder of the unaffected hemibody 23,75 cm^2^	tcDCS: Controlesional cerebellar hemisphere (O1 or O2 position) 12,56 cm^2^ tDCS: Ipsilesional orbit 12,56 cm^2^ tsDCS: D10 spinous process 23,75 cm^2^	tcDCS 2 mA tDCS 2 mA tsDCS 2.5 mA	20 min During training	Group 1: cathodal tcDCS+cathodal tsDCS Group 2: anodal tDCS+cathodal tsDCS
Picelli et al. (31)	Starstim®, (Neuroelectrics, Spain)	tcDCS:buccinator muscle 12,56 cm^2^ tsDCS: shoulder of the unaffected hemibody 23,75 cm^2^	tcDCS: cerebellar hemisphere (O1 or O2 position) 12,56 cm^2^ tsDCS: D10 spinous process 23,75 cm^2^	tcDCS 2 mA tsDCS 2.5 mA	20 min During training	Group 1: contralesional cathodal tcDCS+cathodal tsDCS Group 2: ipsilesional cathodal tcDCS+cathodal tsDCS
Seo et al. (30)	DC-Stimulator Plus (NeuroConn GmbH, Germany)	CMA presumed controlling affected leg (lateral Cz position) 35 cm^2^	Forehead above the contralateral orbit 35 cm^2^	2 mA	20 min Before training	Group 1: anodal tDCS Group 2: sham tDCS

*CMA, cortical motor area*.

### Comparison

In the studies selected for the current systematic review, only three studies used a RCT sham controlled study design (20, 22, 30). Three studies are methodological studies, in which randomization was used to test different stimulation sites (i.e., cerebellum or spinal cord) or different types of stimulation (anodal and cathodal) (29, 31, 32). In the last study (28), a comparison was performed between the effects of NIBS delivered before, during, or after RAGT.

### Outcome

In two out three studies that compared real stimulation (anodal on M1) and sham stimulation (supraorbital stimulation), a significant clinical improvement was found in the real group with respect to the sham group. Danzl et al. (20) found a statistical difference (time x group interaction) in the FAC, TUG, and SIS-16 score evaluated before and immediately after training. Seo et al. (30) found a statistical improvement in the real group in the FAC and 6MWT score at a 4-week follow-up. Geroin et al. (22) reported only a TIME effect between the two groups without time x group interaction (real vs. sham) (Table 2).

Methodological studies have shown a significantly greater improvement in a walking capacity recorded with 6MWT. Picelli et al. (29) find a statistical improvement in groups that underwent anodal tDCS + cathodal spinal stimulation (tsDCS) during RAGT with respect to anodal tDCS or cathodal tsDCS alone. In Picelli et al. (32), a significant improvement was found in the group undergoing a cathodal cerebellar stimulation plus cathodal tsDCS with respect to the patients that underwent anodal tDCS plus cathodal tsDCS. Differently, no difference has been reported by two different protocols of cerebellar transcranial direct current stimulation combined with transcutaneous spinal direct current stimulation on RAGT (31) and in the comparison of dual-site direct current stimulation (dstDCS) performed before, during, and after RAGT (28).

All the studies performed the clinical evaluation before, after treatment, and in the post-intervention follow-up. Time between the end of the treatment and the follow-up ranged between 2 weeks (29, 31, 32) and 3 months (28). More than half of the included studies (28, 29, 31, 32) had outcome measurements at multiple time points, up to 3 months after the intervention. All the studies used the functional ambulatories category (FAC), of which two used it as primary outcome measure (28, 30). The 6-meter walking test (6MWT) was assessed in 5 out of seven studies (22, 28, 29, 31, 32) as a primary outcome, while Seo et al. (30) used it as a secondary outcome. More than a half of the studies (22, 29, 31, 32) analyzed spatiotemporal gait parameters as a secondary outcome. Other scales that were frequently used were Motricity Index leg-subscore (22, 28, 29, 31, 32), 10-meter walking test (10MWT) (18, 20,26;, 28), and Ashworth scale (29, 31, 32). Outcomes that were less considered were Berg Balance Scale (BBS), Fugl-Meyer Assessment of Lower Extremity (FMA-LE), Medical Research Council (MRC) Scale, Modified Ashworth Scale (MAS), Rivermead Mobility Index, Stroke Impact Scale 16 (SIS-16), Timed Up and Go (TUG), Functional Independence Measure (FIM), and Tinetti Scale. Additionally, Naro et al. (28) investigated the ratio between the motor-evoked potential (MEP) of the affected and unaffected hemisphere to estimate interhemispheric balance inhibition. Table 5 shows the results of the studies.

**Table 5:** Results of the studies.

	**Primary outcomes**	**Secondary outcomes**
**Study**		
Danzl et al. (20)	**10MWT**: no significant, but results favored the active tDCS group (*p* = 0.19)	**TUG**: significant improvement in the active tDCS group compared to the sham group (*p* = 0.066) **BBS**: no significant improvement in the active tDCS group compared to the sham group (*p* = 0.919) **FAC**: significant improvement in the active tDCS group compared to the sham group (*p* = 0.028) **SIS-16**: significant improvement in the active tDCS group compared to the sham group (*p* = 0.062)
Geroin et al. (22)	**6MWT**: significant improvement in group 1 and 2 compared to group 3 at T1 and T2. No significant difference between group 1 and 2 **10MWT**: significant improvement in group 1 and 2 compared to group 3 at T1 and T2. No significant difference between group 1 and 2	**Spatiotemporal gait parameters**: no significant differences between group 1 and 2, in both T1 and T2 evaluations. Significant difference between group 1 and 2 compared to group 3 at T1 and T2 evaluations. **FAC**: significant improvement in groups 1 and 2 compared to group 3 at T1 and T2 evaluations. No significant differences between group 1 and 2, in both T1 and T2 evaluations. **R-MI**: significant improvement in groups 1 and 2 compared to group 3 at T1 and T2 evaluations. No significant differences between group 1 and 2, in both T1 and T2 evaluations. **MI**: significant improvement in groups 1 and 2 compared to group 3at T1 and T2 evaluations. No significant differences between group 1 and 2, in both T1 and T2 evaluations. **MAS**: not reported
Naro et al. (28)	**10MWT**: no significant changes **6MWT**: significant difference between the treatmentsover time (*p* < 0.001) in relation to on-RAGTand post-RAGT **FIM**: improvement obtained over time was similar for all groups (*p* < 0.001) **Tinetti Scale**: significant difference between the treatmentsover time (*p* < 0.001) in relation to on-RAGTand post-RAGT **MI**: improvement obtained over time was similar for all groups (*p* < 0.001) **FAC**: significant difference between the treatmentsover time (*p* < 0.001) in relation to on-RAGTand post-RAGT **Ratio between the MEP of the affected and unaffected hemisphere**: MEPaff/unaff ratio was always lower than 1. MEP ratio influenced the dstDCS outcome (F = 9.6, p < 0.001) with regard to on-RAGT (*p* < 0.001).	–
Picelli et al. (29)	**6MWT**: significant differences in walking distance between the groups at the T1–T0 (*p* = 0.014) and T2–T0 (*P* = 0.005) evaluations. No significant difference between the groups at the T3–T0 evaluation (*P* = 0.649). Significant differencein group 3 vs. group 1 at T1–T0 (*P* = 0.015) and atT2–T0 (*P* = 0.001) evaluations, as well as in group 3 vs. group 2 at T1–T0 (*P* = 0.010) andT2–T0 (*P* = 0.015) evaluations. No significant difference in group 2 vs. group 1 results.	**FAC**: no significant difference between the groups at the T1–T0 (*P* = 0.126), T2–T0 (*P* = 0.368) and T3–T0 (*P* = 0.342) evaluations. **MI**: no significant difference between the groupsatthe T1–T0 (*P* = 0.107), T2–T0 (*P* = 0.355) and T3–T0 (*P* = 0.715) evaluations. **AS**: no significant difference between the groups at the T1–T0 (*P* = 0.312), T2–T0 (*P* = 0.259), and T3–T0 (*P* = 0.259) evaluations. **Cadence**: significant differences between the groups at the T1–T0 (*P* = 0.003) and T2–T0 (*P* = 0.016) evaluationsbut not at the T3–T0 evaluation (*P* = 0.405). Significant difference in group 3 vs.group 1 results at T1–T0 (*P* = 0.002) and T2–T0 (*P* = 0.013) evaluations, as well as in group3 vs. group 2 results at T1–T0 (*P* = 0.005) and T2–T0 (*P* = 0.016) evaluations. No significantdifference in group 2 vs. group 1 **Ratio between single and double support duration**: no significant difference between the groups at T1–T0 (*P* = 0.512), T2–T0 (*P* = 0.416), and T3–T0 (*P* = 0.220) evaluations
Picelli et al. (29)	**6MWT**: significant differences in walking distance between the groups at the T1–T0 (*P* = 0.041). No significant difference betweengroups at T2–T0 (*P* = 0.650) and T3–T0 (*P* = 0.545).	**FAC**: no significant difference between the groups at T1–T0 (*P* = 1.000), T2–T0 (*P* = 1.000) and T3–T0 (*P* = 0.317). **MI**: significant difference between the groupsatT1–T0 (*P* = 0.017), T2–T0 (*P* = 0.045) and T3–T0 (*P* = 0.008). **MAS**: no significant difference between the groups at T1–T0 (*P* = 0.210), T2 (*P* = 0.251) and T3 (*P* = 0.644) **Cadence**: significant differences between the groups at the T1–T0 (*P* = 0.019) but not at T2–T0 (*P* = 0.650) and T3–T0 (*P* = 0.545). **Ratio between single and double support duration**: no significant difference between the groups at T1–T0 (*P* = 0.472), T2–T0 (*P* = 0.212), and T3–T0 (*P* = 0.075) evaluations
Picelli et al. (31)	**6MWT**: no significant difference between the two groups at T1 (*P* = 0.976), T2 (*P* = 0.178) and T3 (*P* = 0.069).	**FAC**: no significant difference between the groupsat T1 (*P* = 0.565), T2 (*P* = 0.538) and T3 (*P* = 0.711) **MI**: no significant difference between the groupsat T1 (*P* = 0.854), T2 (*P* = 0.854) and T3 (*P* = 0.806) **MAS**: no significant difference between the groupsat T1 (*P* = 0.720), T2 (*P* = 0.845) and T3 (*P* = 0.721) **Cadence**: no significant difference between the groupsat T1 (*P* = 0.378), T2 (*P* = 0.635) and T3 (*P* = 0.778) **Ratio between single and double support duration**: no significant difference between the groupsat T1 (*P* = 0.867), T2 (*P* = 0.715) and T3 (*P* = 0.666)
Seo et al. (30)	**FAC**: significant greater improvement in the Anodal groupthan in the Sham group at T2 (66.7% vs. 12.5%, p = 0.024)	**10MWT**: no significant difference between T1 and T0 between the groups **6MWT:** no significant difference between T1 and T0 between the groups **BBS**: no significant difference between T1 and T0 between the groups **FMA-LE**: no significant difference between T1 and T0 between the groups **MRC**: no significant difference between T1 and T0 between the groups **MEP**: no significant difference between the groups

*6MWT, 6-min walking test; 10MWT, 10-meter walking test; AS, Ashworth scale; BBS, Berg balance scale; CL, controlesional; FAC, functional ambulatories category; FIM, funtional indipendence measure; FMA-LE, Fugl-Meyer assessment of lower extremity; IL, ipsilesional; JM, joint mobilization; LL, lower limb; MAS, modified Ashworth scale; MEP, motor-evoked potentials; MI leg subscore, motricity index leg subscore; MRC, medical research council scale; R-MI, rivermead motricity index; SIS-16, stroke impact scale 16; ST, spatiotemporal, tDCS, transcranial direct current stimulation; tsDCS, transpinal direct current stimulation; TUG, timed up and go*.

### Methodological Quality

Methodological quality was assessed with RoB-2 (26) for all the studies except one (28). As regards to the studies that compared real versus sham stimulation (18, 20, 28), the randomization process showed some concerns in one study (20) that did not report the random generation method. All the other biases were judged as “low risk.”

**Figure 2:**
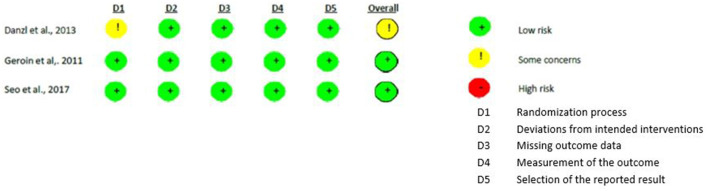
Assessment of Risk of Bias in real versus sham studies.

**Figure 3:**
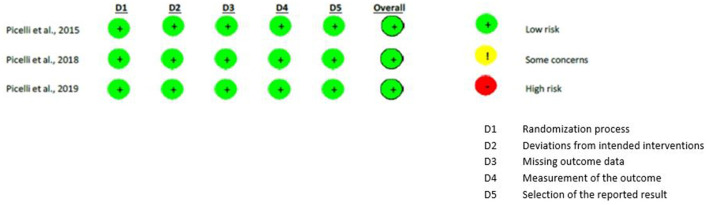
Assessment of Risk of Bias in methodological studies.

Differently, all the risks of bias of the methodological studies (29, 31, 32) were judged as “low risks.” Figures 2, 3 show the assessment of the risks of bias with the selected studies.

Naro et al. (28) study was evaluated using the MINORS individual score, and its final rating was 19 over 24. “The follow-up period appropriate to the aim of the study” and “loss to follow-up <5%” did not get a maximum score, while “prospective calculation of the study size” received the minimum score. Details of the MINORS score are reported in Table 6.

**Table 6:** Individual MINORS score.

	**Naro et al. (28)**
Clearly stated aim	2
Inclusion of consecutive patients	1
Prospective collection of data	2
Endpoints appropriate to the aim of the study	2
Unbiased assessment of the study endpoint	2
Follow-up period appropriate to the aim of the study	1
Loss to follow up less than 5%	1
Prospective calculation of the study size	0
*Additional criteria in the case of comparative study*	
An adequate control group	2
Contemporary groups	2
Baseline equivalence of groups	2
Adequate statistical analyses	2
TOTAL SCORE	19

*0, not reported; 1, reported but inadequate; 2, reported and adequate*.

## Discussion

Restoring the ability to walk is the main aim of post-stroke rehabilitation; stroke survivors commonly present reduced ability to walk and limited activities inside and outside their home. Walking has been described to have a greater chance of post-stroke recovery than hand function because it is less dependent on the post-lesion integrity of the corticospinal tract. Although it requires a lower degree of residual motor function after stroke, gait performance often persists impaired in patients with chronic stroke due to decreased dorsiflexors strength and altered interaction between different connected functional networks involved in walking (31). The present systematic review investigated the effects of combining non-invasive brain stimulation with robot-assisted therapy for gait recovery in patients with chronic stroke.

It has been shown that NIBS techniques are able to harness brain plasticity (14), and there are several neurostimulation techniques and clinical applications, both open-loop and closed-loop, which seem to support cerebral neuroplasticity (34); the most studied and used are tDCS and TMS. While researchers have shown that tDCS has the potential to improve upper extremity motor recovery following stroke if paired with intensive motor training, only a few studies have examined the effects of tDCS on lower extremity motor function (20). This systematic review suggests that tDCS of the leg area of the motor cortex in the impaired hemisphere or cerebellar transcranial direct current stimulation (tcDCS) over the contralesional/ipsilesional cerebellar hemisphere combined or not with transcutaneous spinal direct current stimulation (tsDCS) and in addition to RAGT produce an improvement in walking function, in particular regarding walking ability, as measured by FAC, and walking capacity, as measured by 6MWT.

What can be for tDCS and RAGT the correct dosage, intensity, duration, order of application, and, moreover, for tDCS, type of stimulation and the site remains the subject of further studies.

There are few studies that use NIBS in association with RAGT. tDCS is among the NIBS elective technique of neurostimulation used in all clinical trials in combination with RAGT. Five out of seven studies, included in this review, used tDCS treatment protocol, which consisted in the stimulation of the motor cortex for 20 min, 5 days a week, for 2 weeks; despite this, it has not yet been determined the best stimulation site (i.e., affected or contralesional hemisphere) and the best timing of stimulation in patients with stroke (35, 36). Indeed, there was no difference whether tDCS was administered before, during, or after the robotic therapy (28). This contrasts with the results found in a study combining tDCS with cognitive exercises that showed improvement in the execution times of the proposed exercises only when the tDCS was performed during training execution (37). Further studies are needed to clarify what is the right timing of stimulation during RAGT.

Furthermore, this systematic review highlighted the need to clarify whether the combination of different sites of tDCS and spinal stimulation can enhance the effects of RAGT; several studies suggest that the stimulation of the nervous system at multiple sites might result in a functional improvement in patients with stroke (e.g., paired associative stimulation—PAS of peripheral and central nervous system) (34). Because the central nervous system (CNS) controls both walking pattern generation and descending control from brain, methods aimed at promoting both spinal and supraspinal activities have been recommended in patients with stroke in order to retrain walking (38). It is plausible that combined supraspinal and spinal stimulation is needed to obtain significant additional effects on RAGT. Thoracic cathodal tsDCS was found to improve motor unit recruitment in healthy people (39). Depending on the topography of spinal cells and the current direction, thoracic cathodal tsDCS should make motoneurons more responsive to synaptic activation but less prone to generate spontaneous activity that inhibits interneuronal networks (39); this could produce positive spasticity control effects, but, furthermore, neurophysiological analyses are required to clarify the effects of tsDCS on muscle overactivity (29) and to investigate both the specific timing in which it is applied and to clarify what the specific factors are that influence its effectiveness. (e.g., state of the brain and spontaneous neuronal activity) (34).

Future pieces of research will have to clarify the role of the combination of TMS with RAGT and the cerebellum implication in stroke recovery; the cerebellum is known to be strongly implicated in the functional reorganization of motor networks in patients with stroke, especially for gait and balance functions. Koch et al. (19) have demonstrated that cerebellar intermittent θ-burst stimulation promotes gait and balance recovery in patients with stroke by acting on cerebello-cortical plasticity. The patients were randomly assigned to treatment with CRB-iTBS or sham iTBS applied over the cerebellar hemisphere ipsilateral to the affected body side immediately before physiotherapy daily, during 3 weeks. The patients treated with CRB-iTBS, but not with sham iTBS, showed an improvement of gait and balance functions, as revealed by a pronounced increase in the mean (SE) Berg Balance Scale score. The patients treated with CRB-iTBS, but not sham iTBS, showed a reduction of step width at the gait analysis and an increase of neural activity over the posterior parietal cortex.

From Wessel et al. (40) in pieces of research, the cerebellum provides unique plasticity mechanisms and has vast connections to interact with neocortical areas. Moreover, the cerebellum could serve as a non-lesioned entry to the motor or cognitive system in supratentorial stroke.

Finally, papers in which a robotic treatment of the lower limb is associated with non-invasive brain stimulation have been few to date for a series of considerations that arise from literature and clinical experience: (1) not all rehabilitation centers have available exoskeletal robots and non-invasive brain stimulation techniques; (2) necessary personnel trained in the use of robots and NIBS; (3) the need for time, space, and human resources; (4) the need for broad and long-term patient compliance.

It would be important to compare more homogenous rehabilitation protocols to better appreciate their beneficial effects on post-stroke recovery. Moreover, considering that each stroke patient is unique in his/her characteristics, it would be probably better to design a therapeutic intervention tailored on every single patient (34).

Given the limited number of studies, the heterogeneity in the treatment protocol and the outcome assessment techniques, it was not possible to carry out a meta-analysis to obtain a quantitative summary of the results. We have found some limitations that may present challenges for future research: sample size and few RCT studies, no neurophysiological assessment with transcranial magnetic stimulation (TMS) was performed to assess cortical excitability and brain connectivity before and after treatments, the lesion site as cortical and subcortical has not been taken into account, sometimes, it was difficult to identify the precise injury extension (heterogeneous properties of stroke), finally, generalization of the stimulation protocols (30). Moreover, no short follow-up nor comparison was done with other non-invasive brain stimulation techniques, and the studies have included only patients with chronic supratentorial ischemic stroke, and we cannot draw conclusions about the effects of the current protocols of NIBS on RAGT in patients with other conditions, as acute or subacute supratentorial ischemic stroke, hemorrhagic, or cerebellar stroke.

The studies' data support the hypothesis that anodal tDCS, combined with thoracic cathodal tsDCS, may be useful to improve the effects of RAGT in patients with chronic stroke. Moreover, cerebellum NIBS could represent a promising interventional strategy to improve residual motor functions and recovery after stroke, modulating cerebellar brain inhibition and facilitating motor skill relearning. Finally, no adverse events were recorded during the study (31).

## Conclusion

The current systematic review showed a positive effect on walking recovery of combination of robot-assisted gait training with non-invasive brain stimulation. Specifically, the use of 20 min of tDCS (1.5–2 mA), 5 times/week for 2 weeks, can increase gait skills in patients with chronic stroke. Heterogeneity was found on the site of stimulation, the type of robot device (end effector, exoskeleton), and stimulation protocol with respect to robot-assisted therapy (before, online, or after). Future RCTs are needed to further validate the findings of these pieces of research.

## Data Availability Statement

The original contributions presented in the study are included in the article/Supplementary Material, further inquiries can be directed to the corresponding author/s.

## Author Contributions

FB, MB, and GM: conceptualization. MB, FS, BC, LC, and AM: writing—original draft preparation. MB, LC, BC, and AM: methodology. BC, AM, GM, VD, FB, LZ, and SS: writing—review and editing. SS, VD, SP, LZ, and FB: supervision. All authors have read and agreed to the published version of the manuscript.

## Conflict of Interest

The authors declare that the research was conducted in the absence of any commercial or financial relationships that could be construed as a potential conflict of interest.

## Publisher's Note

All claims expressed in this article are solely those of the authors and do not necessarily represent those of their affiliated organizations, or those of the publisher, the editors and the reviewers. Any product that may be evaluated in this article, or claim that may be made by its manufacturer, is not guaranteed or endorsed by the publisher.
