# Supplementary material for: Combining Robot-Assisted Gait Training and Non-Invasive Brain Stimulation in Chronic Stroke Patients: A Systematic Review
Source: Front Neurol. 2022 May 2;13:795788. doi: 10.3389/fneur.2022.795788 (PMC9108455; doi:10.3389/fneur.2022.795788)
Supplement: Supplementary file 1 [file Table_1.DOCX]

**Appendix 1. Search strategy**

| Literature search (last search performed March 15, 2021) | |
| --- | --- |
| PubMed | (stroke[MeSH Terms]) AND ((Exoskeleton Device[MeSH Terms]) OR (Robotic Exoskeleton[MeSH Terms]) OR (robot-assisted)) AND ((magnetic stimulation, transcranial[MeSH Terms]) OR (Transcranial Direct Current stimulation[MeSH Terms])) |
| Scopus | ( ALL ( stroke )  AND  ALL ( robot-assisted )  AND  ALL ( exoskeleton )  AND  ALL ( transcranial  AND  magnetic  AND  stimulation )  OR  ALL ( transcranial  AND  direct  AND  current  AND  stimulation ) |
| EMBase | ('stroke'/exp OR stroke) AND (('exoskeleton'/exp OR exoskeleton) AND ('device'/exp OR device) OR (robotic AND ('exoskeleton'/exp OR exoskeleton)) OR 'robot assisted') AND (magnetic AND stimulation, AND transcranial OR (transcranial AND direct AND current AND ('stimulation'/exp OR stimulation))) |
| EBSCO | chronic stroke AND (exoskeleton OR robot-assisted) AND (transcranial magnetic stimulation or tms or rtms OR transcranial direct current stimulation or tdcs) |
| PedRO | “Stroke” AND “Exoskeleton” |
| Web Of Scince | ALL=chronic stroke  AND ALL=(exoskeleton  OR robot)  AND ALL=(transcranial magnetic stimulation  OR tms  OR rtms  OR transcranial direct current stimulation  OR tdcs) |
